# Ultrastructural Aspects of Physiological Mineralization: A Comparative Study in Different Hard Tissues

**DOI:** 10.3390/biom15070932

**Published:** 2025-06-26

**Authors:** Marina Borgese, Mario Raspanti, Marina Protasoni, Piero Antonio Zecca, Fulvia Ortolani, Marcella Reguzzoni

**Affiliations:** 1 Department of Medicine and Technological Innovation, Insubria University, 21100 Varese, Italy; marina.borgese@uninsubria.it (M.B.); marina.protasoni@uninsubria.it (M.P.); pieroantonio.zecca@uninsubria.it (P.A.Z.); marcella.reguzzoni@uninsubria.it (M.R.); 2 Department of Medicine, University of Udine, 33100 Udine, Italy; fulvia.ortolani@uniud.it

**Keywords:** calcification, collagen, cartilage, dentin, calcifying tendon, SEM

## Abstract

The calcified tissues of vertebrates are essentially represented by bone, cartilage, dentin and calcified tendons. In all these tissues a major hallmark of mineralization is the deposition of the inorganic phase on a pre-existing collagen template, but evident differences exist among these materials and the molecular details of the process are still incompletely understood. In this study, the ultrastructural aspects of the mineral phase of these tissues were investigated by means of high-resolution scanning electron microscopy (HR-SEM) after low-temperature thermal deproteination, a technique allowing a direct, unrestricted visualization of the mineral component. Each tissue showed distinctive features. In most cases, calcification proceeds in a discontinuous way through the formation of clumps or clusters of mineralized tissue; in all cases, except cartilage, the mineral phase shows an evident relationship with the layout and/or the D-period of the collagen fibrils. Our results highlight the peculiar aspect of the mineralization process in the cartilage with respect to the other tissues, all of them containing collagen type I instead of type II, and suggest that a different molecular mechanism may be at work. It is still unclear whether and how this may be related to the content, exclusive of cartilage, of collagen type II. The identification of the tissue-specific features exhibited by cartilage versus those shared by all the other three tissues, although from different species, requires further research on physiological calcification.

## 1. Introduction

Biomineralization is a complex process which involves several tissues in many ways. In vertebrates, physiological mineralization is essentially confined to connective tissues—the only notable exceptions being the otoconia in the inner ear, the teeth enamel and the eggshell of birds. Most studies have been carried out on bone, which is indisputably the most important calcified tissue from both the quantitative and functional viewpoints. Other important calcified connective tissues are the dentin, cartilage and calcifying tendons of birds. In all these tissues, the process includes inorganic infiltration of a pre-existing collagenous scaffold.

Despite the progress made by recent research, several molecular details of mineralization are still incompletely understood [1]. There is now some evidence that mineral precipitation occurs spontaneously when the relevant inorganic ions (Ca and P) are available and inhibitors are not present [2,3,4], but several factors (and their interaction) can control the inception and the extent of the mineralization. The outcome of the process can be very different in different tissues.

Some decades ago, the diverse aspects observed led to the recognition that collagen fibrils and matrix vesicles, released by osteoblasts, chondroblasts, odontoblasts or tenocytes, may both serve as independent nucleation centers for mineral deposition, but with very different features [5]. Matrix vesicles are released into the interfibrillar spaces as small, membrane-bound vesicles adhering to collagen fibrils by means of specific annexins. Several ion channels and enzymes are attached to their membrane including ectonucleotide pyrophosphatase/phosphodiesterase 1 (ENPP1), tissue nonspecific alkaline phosphatase (TNAP), calcium channel proteins (Anx), and phosphate transporters (Pit1). Exogenous inorganic ions are transferred by Annexin V and Pit1 in the vesicle interior, where they form mineral particles that eventually burst the membrane and grow in the interfibrillar space into fractal-like, micron-sized globular particles [5,6]. Similar extrafibrillar mineral deposits have also been observed in pathological calcifications [7,8,9], with the substantial differences that here the ECM is involved only secondarily, with cell debris being the primary structures involved in early mineralization [10]. Cell debris may result from various types of programmed cell death including apoptosis, necroptosis, pyroptosis, and ferroptosis as reported for vascular calcification [11], or a further type of an uncoded pro-calcific cell death associated with intracytoplasmic release of acidic lipid material. This and its by-products can act as calcium salt nucleators, as reported for aortic valve interstitial cells stimulated with pro-calcific media in vitro [12] as well as in actual calcific aortic valve stenosis [13].

On the other hand, mineralization can also occur in the absence of matrix vesicles by enzymatic removal in the extracellular space of several tissue-specific inhibitors such as pyrophosphate (PPi), osteopontin (OPN), osteoprotegerin, secreted phosphoprotein 1 (SPP1), Gla proteins (BGLAP and MGP), and α2-HS glycoprotein (AHSG) [3,14]. Under these conditions, tiny needle-shaped mineral particles appear simultaneously on a large number of individual unrelated collagen fibrils. Electron microscopic tomography studies demonstrated beyond any doubt that the first deposition of calcium salts takes place at specific sites within the gap zone of collagen fibrils, in both bone [15,16,17] and calcifying tendons [18,19,20]. Subsequently, as the particles grow in number and length, the collagen fibrils swell, lose their individuality and merge [21] as the process extends to the interfibrillar space [17,22]. More recent high-resolution 3D imaging studies showed that, on a larger scale, the collagen-driven mineralization proceeds by the formation of individual clusters of mineralization, or *tesselles,* at least in bone [23,24,25] and tendon [23].

While these findings undoubtedly question the exclusive role once ascribed to the matrix vesicles in the mineralization process, they did not rule out their involvement and they still are actively studied [26,27,28,29,30,31,32,33]. Globular bodies reminiscent of those produced by matrix vesicles have been recently observed in the costal cartilage [34], in the articular cartilage [35] and the growth plate [36,37]. These data seem to suggest that, although bone and cartilage form an integrated functional unit [38,39], a different form of mineralization is at work in cartilage with respect to bone and possibly to dentin and calcifying tendons.

Not all morphological techniques are suitable for ultrastructural research on mineralized tissues. Light microscopy, like other techniques such as confocal laser scanning, second harmonic generation or Fourier transform infrared, does not have adequate lateral resolution for true ultrastructural research. Transmission electron microscopy (TEM) has adequate resolution but still requires prior decalcification; and, like all sectional techniques, can only represent the boundary between soft and mineralized matrix, which is intrinsically two or three dimensional, as a single line. Micro-computed tomography can provide really three-dimensional data but its lateral resolution is still limited.

The present research was carried out by means of high-resolution scanning electron microscopy (HR-SEM) in order to image the ultrastructure of the mineral phase of these four tissues. The mineralization process may not be the same in them all, and a better understanding of the morphology of the mineral crystals would help determine the process by which they form in different contexts.

## 2. Materials and Methods

Samples of articular cartilage and of its subchondral bone were obtained at the abattoir from the femoral head of two 1-year-old steers, and samples were taken of mineralized cartilage and compact bone from the subchondral plate. Similarly, samples of calcifying gastrocnemius tendon were obtained at the abattoir from three adult turkeys. Two human permanent teeth were extracted for orthodontic reasons from healthy individuals of either sex. All the specimens were immediately dissected, debrided from the surrounding tissues, reduced if necessary and fixed overnight in Karnovsky fixative in 0.1 M Na-cacodylate buffer.

All specimens were frozen in solidifying butane at −138 °C, fractured under liquid nitrogen at −196 °C in order to obtain clean, uncontaminated surfaces and subsequently dehydrated with graded ethanol and hexamethyldisilazane. The specimens were then thermally treated at 350 °C for 24 h, a process able to completely remove the soft matrix while not affecting the mineral phase [40].

All samples were then mounted on appropriate stubs with colloidal silver, sputter-coated with gold-palladium and observed with a Zeiss Gemini 360 FEG-SEM (Carl Zeiss, Oberkochen, Germany). In total, over 300 pictures were taken of the four tissues. All images were acquired in digital form as uncompressed TIFF files of 2048 × 1536 pixels with no further processing.

## 3. Results

At the temperature used in this study, the thermal treatment removes all the soft matrix, leaving behind only the mineral phase, which forms a sort of undisturbed cast of the mineralized fibrils. This technique has been shown not to affect the size, shape and layout of the original fibrils while allowing an unobstructed visualization of the mineral. In the following text, the term “collagen fibrils” will be used for brevity to designate these casts.

All the tissues studied exhibited a specific, peculiar morphology.

**Figure 1:**
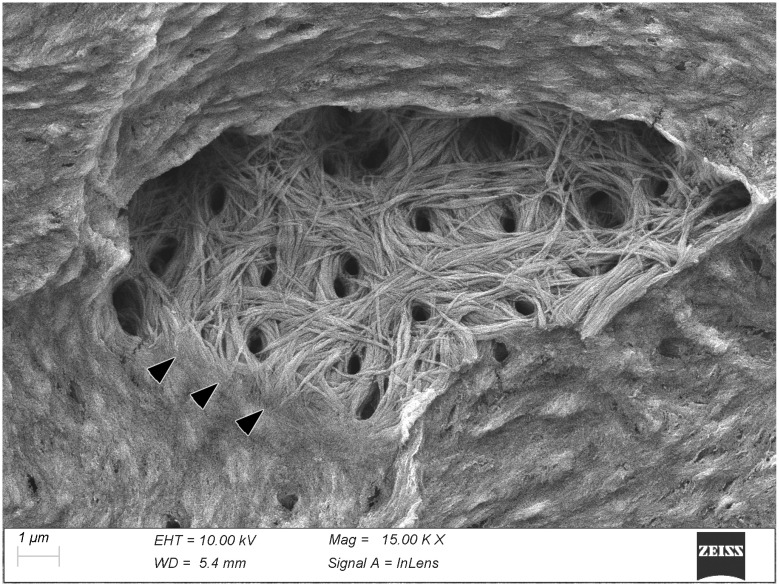
Thermally deproteinated bone. The fracture surface intersects an osteocytic lacuna and reveals on its surface the web-like arrangement of the mineralized fibrils around the openings of the canalicular system. Near the lower edge of the lacuna the fibrils can be seen to merge and disappear into the compact bone matrix (arrowheads).

Bone was perhaps the most difficult tissue to image at high resolution because its matrix is very compact and only its natural surfaces, such as the interior of the osteocyte lacunae, allow a clear visualization of individual fibrils that otherwise appear essentially fused together (Figure 1).

**Figure 2:**
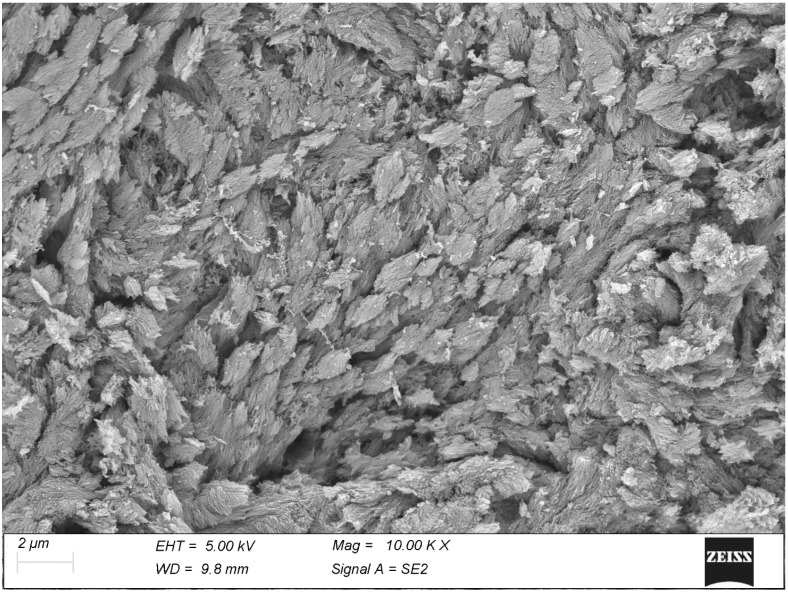
The mineralizing surface of the bone reveal the mineralization pattern, which takes place through the development of spindle-shaped *tesselles*.

**Figure 3:**
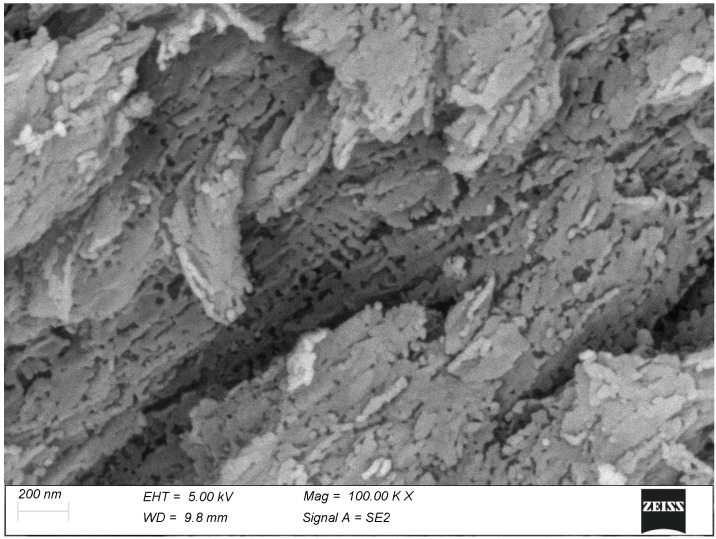
A higher magnification of bone shows multiform plate-like mineral particles. Their close relationship with the collagen D-period is now more evident.

The mineralization process often proceeds unevenly with the formation of spindle-shaped clumps of calcifying fibrils, easily recognizable once the interposed soft matrix has been vaporized by the thermal treatment (Figure 2). The mineral particles appear to infiltrate the collagen fibrils, where they maintain the shape and the course of the fibrils and often show an evident relation with the D-period (Figure 3).

**Figure 4:**
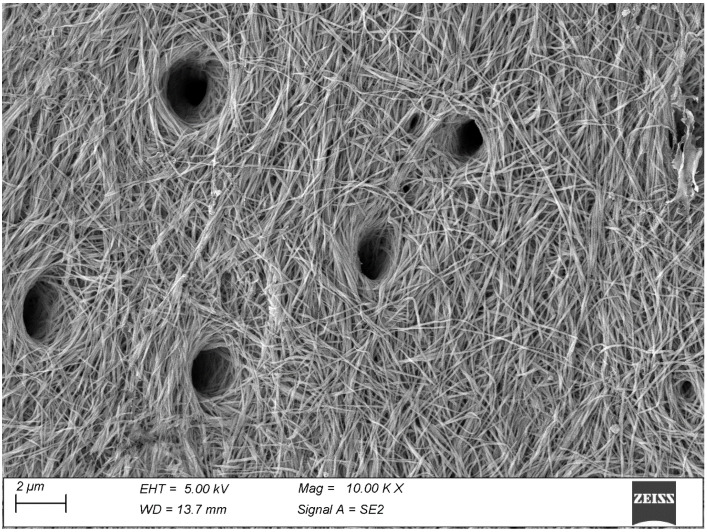
Slender mineralized fibrils weave among the opening of dentinal canals at the predentin/dentin boundary. The fibrils are particularly thin and uniform in this tissue.

**Figure 5:**
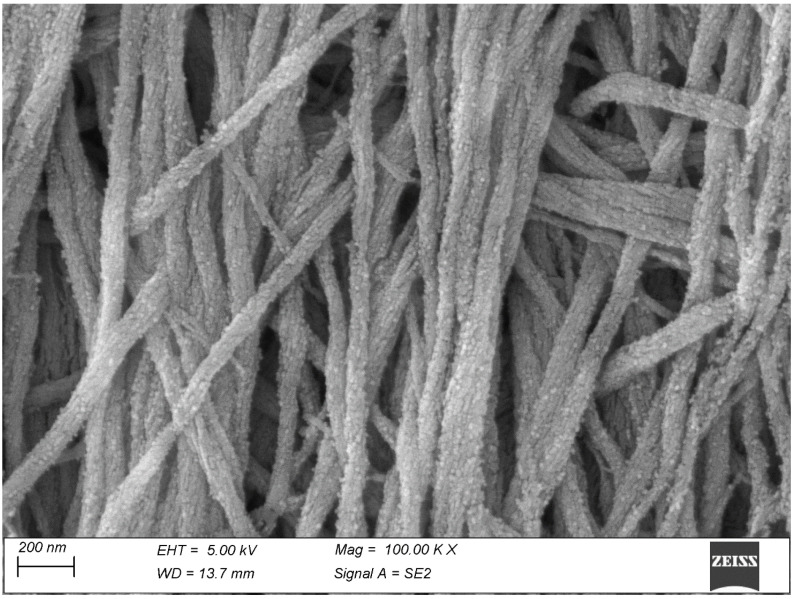
A higher magnification of dentin fibrils, covered by tiny mineral platelets. The fibrils are too slender to show their D-period in these conditions, while some fibrils reveal the course of their subunits.

The dentin is rather reminiscent of bone: its fibrils are somewhat thinner but again, individual fibrils remain visible only on the mineralization front (Figure 4) once the soft predentin has been removed by the thermal treatment. Their average diameter, measured on 100 fibrils from five micrographs taken at 100,000×, is just 73.6 ± 12.0 nm. With the deposition of further dentin the mineral deposition increases and the same fibrils fuse to form a compact mass where individual fibrils are no longer recognizable. At higher magnification, the mineral phase appears made of tiny particles encrusting the surface of the collagen fibrils (Figure 5).

**Figure 6:**
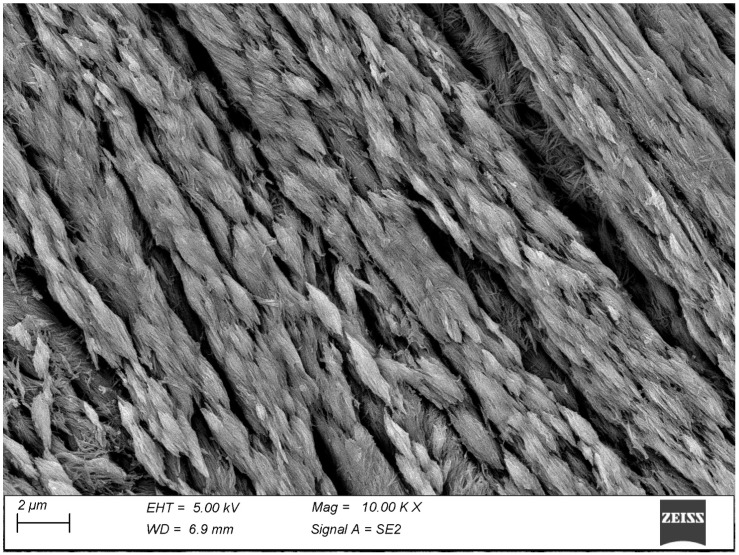
The calcifying surface of tendon confirms the spindle-shaped clusters observed in bone, here even more visible due to the more ordered architecture of this tissue.

**Figure 7:**
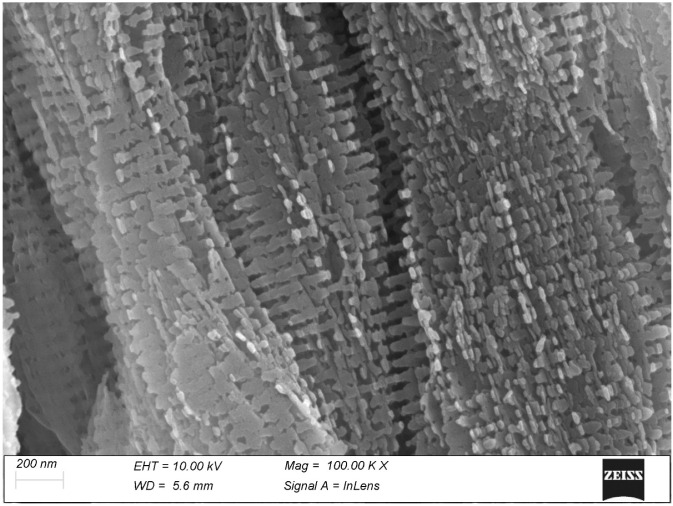
At higher magnification, the mineral phase of the turkey tendon. The mineral plaquettes are rather uneven in shape but their regular 67 nm axial periodicity is clearly evident.

The calcifying tendons of birds represent the most accessible of the tissues studied thanks to the ordered, parallel layout of their collagen fibrils. Here, too, calcification seems to progress with the formation of spindle-shaped clusters of mineralization fibrils reminiscent of those of bone (Figure 6). At higher magnification, the mineral phase takes the form of large, flattened plates whose relationship with the collagen D-period is indubitable (Figure 7).

**Figure 8:**
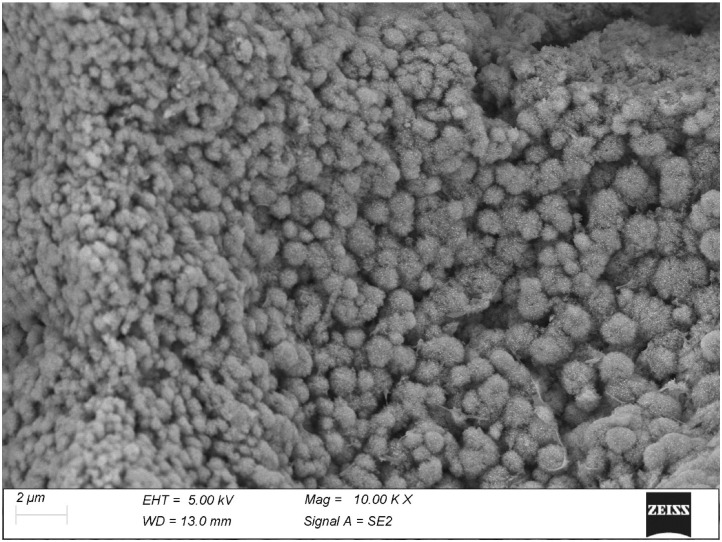
The mineralization front in the depth of articular cartilage. The soft matrix has been removed by the thermal treatment; the mineralization takes place through the appearance of micron-sized isotropic spheroids. Neither an orientation nor a calcified fibril was ever observed.

**Figure 9:**
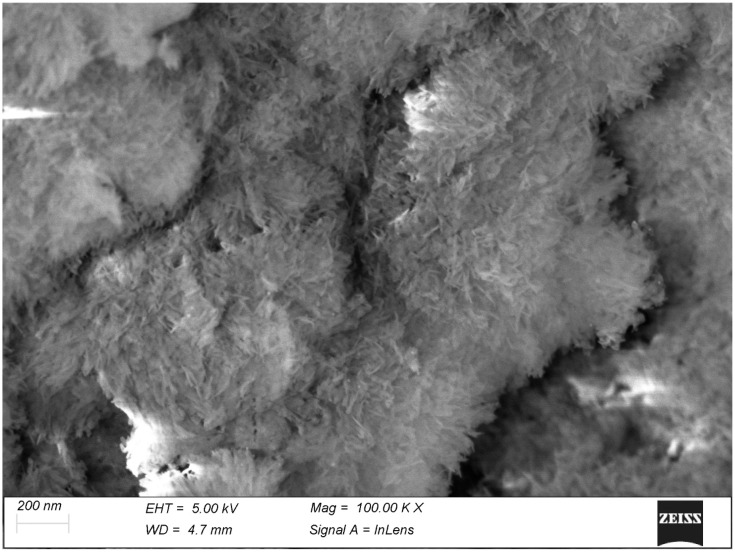
A higher magnification of the mineralized cartilage particles reveals their being made of mineral platelets haphazardly oriented.

The mineralization front of the articular cartilage has been usually seen with histological techniques of radial sections, where it is just visible as an undulating dark line. The thermal treatment of our specimens removed the soft cartilage and exposed the actual mineralization front, making it completely accessible with scanning electron microscopy. Here, the mineralization process takes place by the formation of spheroidal, fractal-like particles (Figure 8), particularly evident where the mineralization is incomplete: either near the mineralization front or, vice versa, deep within the tissue where electrolytes and metabolites must move slowly across the surrounding hard tissues. With the progression of the process these spheroids just become larger until they fuse together into a compact mass. At higher magnification (Figure 9), these spheroids appear made of randomly oriented tiny plaquettes with neither evident order nor orientation. This is the only tissue where no visible alignment was detected within the calcified matrix and where no visible relationship was found between the mineral phase and the layout or the D-period of collagen fibrils.

## 4. Discussion

In both bone and tendon, the onset of mineralization takes place with the formation of ellipsoidal clumps consistent with recent literature data [23,24,25]. It must be noted that the elongated shape of these particles corresponds to the parallel array of the collagen fibrils; it can be hypothesized that if this anisotropy did not exist, the particles would also be isotropic, i.e., spheroidal. The formation of these spheroid/ellipsoids seems to be consistent with a process driven by matrix vesicles but could also be explained by a focal stenciling process of “inhibition of inhibitors” [3]. In both tissues and at both low (10.000×) and high (100.000×) magnification, the mineral particles always show an evident relationship with the collagen fibril D-banding, with the same regular axial periodicity. This is particularly noticeable in the calcified turkey tendon (see Figure 7): the mineral particles are somewhat irregular in shape but their axial spacing can be easily measures by averaging it along several successive periods.

The mineralized fibrils of dentin, which can be only visualized at the predentin/dentin boundary, show a more subtle transformation. Again, individual fibrils are no longer recognizable on the underlying, fully mineralized matrix, so it seems safe to assume that in this tissue, too, the mineral deposition takes place *inside* the fibrils, like in tendon [18], bone [19] and dental cementum [41], as well as *upon* them. The fibrils themselves appear very thin and uniform in diameter, quite different from the plurimodal diameter distribution characterizing tendons. Because of such small size, the collagen D-banding is not visible as it is in the larger fibrils of other tissues. Here, the absence of recognizable ellipsoids suggests a more uniform mineralization process, and points toward an “inhibition of inhibitors” process rather than one driven by matrix vesicles, but the topic is far from being settled [42,43].

By contrast, the cartilage seems to represent a case in itself in that the mineralization here seems totally unrelated to the collagen fibrils. The process takes place through the formation of fractal-like, isotropic spheroids which show no relationship at all with the soft surrounding matrix. Of course these spheroids, while growing, must necessarily come in contact with the surrounding collagen fibrils, but these latter remain not involved in the process since no mineralized fibrils were ever detected in the calcified cartilage. In fact, the mineral clumps may remain spheroidal *exactly* because they bear no relationship with the collagen fibrils, at variance with those of bone and tendon. Observation at high magnification confirms they being made of thin, flat plaquettes randomly oriented. This mineralization pattern is consistent with that described by Christoffersen and Landis [5] as an alternative to the more common collagen-related mineralization found in most tissues.

It is an obvious temptation to connect this difference of mineralization patterns to the different collagen types present in these tissues. It must be noted, however, that no direct cause-effect exists to support this dependence. It has been hypothesized that mineral nucleation within the gap zone of type I collagen fibrils is facilitated by a clustering of highly polar residues [44], but the amino acid sequence (and therefore the polarity distribution) is highly conserved in the helix domains of fibrillar collagens. The differences at the molecular level between collagen types I and II have been the subject of extensive studies [45,46] but no conclusive data have been shown to support this hypothesis.

Nonetheless, two important findings could be *(i)* that the biomolecule Poly(ADP-ribose) (PAR) can act as a nucleator of calcium phosphate apatite crystals forming amorphous calcium-rich spheres which show high affinity for the heterotrimeric collagen fibrils type I at the level of the intraperiod hole zone [47], and *(ii)* that such an interaction seems to occurs at level of distinct binding sites located in register with the intraperiod sub-bands “a” [48], i.e., where C-terminal telopeptides lie, as dictated by the molecular D-period molecular packing. In addition, putative amino acid motifs acting as binding sites for PAR were identified, consisting of coupled arginine and tyrosine residues. It is noteworthy that the C-terminal telopeptides of collagen type II, besides being longer than their counterparts in collagen type I, do not contain any arginine-tyrosine amino acid motifs [46]. Thus, it might be speculated that such differences play a role in the different calcification processes in type II collagen fibrils versus type I.

Moreover, it cannot be excluded that the shorter gap zone characterizing type II collagen fibrils due to the bulkier terminal N- and C-telopeptides may represent a cause or a co-cause, as well as a different distribution of crosslinks and/or a stronger linkage of the fibril-bound proteoglycans, whose persistence might inhibit the mineral deposition. In addition, collagen fibrils type II interact at their surface with the fibril-associated collagen with an interrupted triple helix (FACIT) type IX, which is located along the fibrils in an antiparallel orientation and with its glycosaminoglycan chain sticking out from the fibril surface.

On the other hand, it cannot be ignored that type I collagen fibrils could also have a similar interaction with another member of the FACIT collagen family, type XII, which was found to be nearly homologous to type IX [49]. Thus, none of these factors, even combined, seems to represent structural features justifying the complete absence of mineralized type II collagen fibrils.

In conclusion, the present results reveal the existence of distinct mineralization patterns occurring in four different hard tissues with subtle differences between bone, dentin and mineralized tendon, whereas marked peculiarities are exhibited by mineralized cartilage. Due to the complexity of the various macromolecular components involved, including two genetically distinct fibrillar collagens, type I versus type II, and their still not well-established interactions with associated macromolecules, further studies are required to clarify the mutually exclusive mineral deposition patterns, as clearly indicated by the present three-dimensional ultrastructural study. If we consider the distribution of these tissues, their clinical relevance and, especially in the case of bone and cartilage, that these tissues must continuously transform and adapt to the changing physiological demands they face throughout life, a better understanding of their biomineralization processes certainly seems a worthy objective.

## 5. Conclusions

In bone and turkey tendon, calcification begins with the diffuse formation of ellipsoidal clumps, or *tesselles*, of mineralized collagen fibrils, which, as the process proceeds, swell and merge into a more compact tissue. In tendon and, to a lesser extent, bone, the mineral phase layout shows a clear correspondence with the 67 nm axial period of collagen.

Dentin is made of mineralized fibrils thinner and more uniform than in the locomotory apparatus; they do not form tesselles and their mineral component is represented by tiny particles rather than plaquettes. Possibly due to the small diameter of the dentinal fibrils, their axial period does not remain visible.

Cartilage, by contrast, never shows mineralized fibrils at all. Its mineral phase is represented by fractal-like, micron-sized isotropic mineral aggregates that grow in the interfibrillar space where they eventually merge. The mineralization process appears to take place by a different mechanism with no involvement of the collagen component. It is still unclear to which extent this may be related to the cartilage composition of collagen type II rather than type I, or to other causes.

## Data Availability

Data available from the authors upon request.
